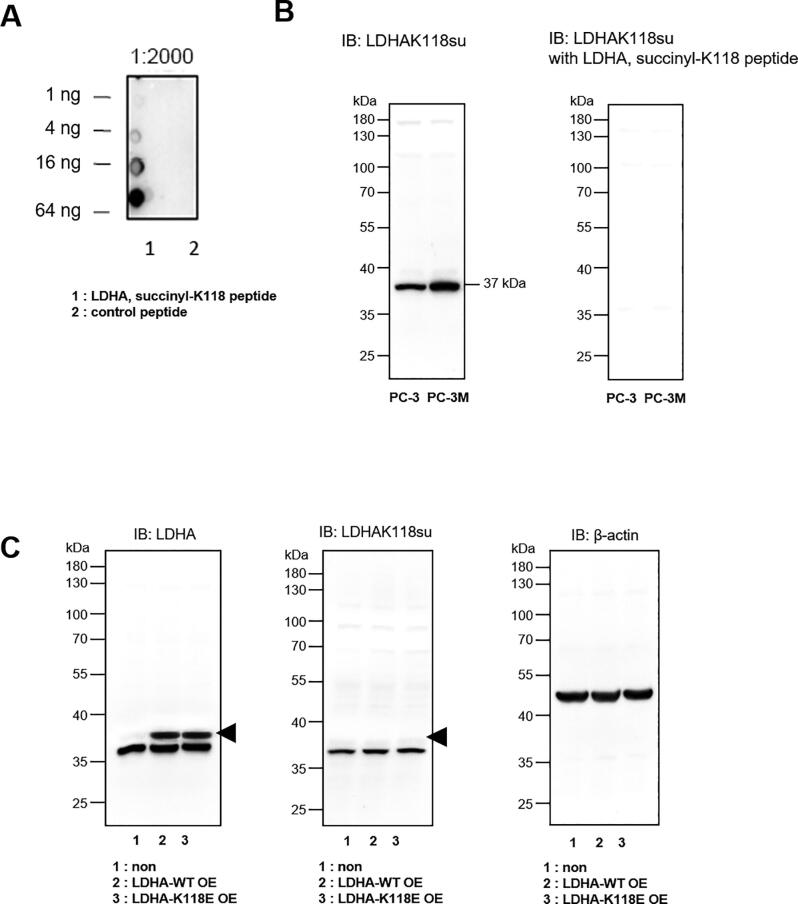# LDHA Desuccinylase Sirtuin 5 as A Novel Cancer Metastatic Stimulator in Aggressive Prostate Cancer

**DOI:** 10.1016/j.gpb.2022.02.004

**Published:** 2022-03-09

**Authors:** Oh Kwang Kwon, In Hyuk Bang, So Young Choi, Ju Mi Jeon, Ann-Yae Na, Yan Gao, Sam Seok Cho, Sung Hwan Ki, Youngshik Choe, Jun Nyung Lee, Yun-Sok Ha, Eun Ju Bae, Tae Gyun Kwon, Byung-Hyun Park, Sangkyu Lee

**Affiliations:** 1 BK21 Plus KNU Multi-Omics Based Creative Drug Research Team, College of Pharmacy, Research Institute of Pharmaceutical Sciences, Kyungpook National University, Daegu 41566, Republic of Korea; 2 Department of Biochemistry and Molecular Biology, Chonbuk National University Medical School, Jeonju, Jeonbuk 54896, Republic of Korea; 3 College of Pharmacy, Chosun University, Gwangju 61452, Republic of Korea; 4 Korea Brain Research Institute, Daegu 41068, Republic of Korea; 5 Department of Urology, School of Medicine, Kyungpook National University, Daegu 41566, Republic of Korea; 6 Department of Urology, Kyungpook National University Hospital, Daegu 41566, Republic of Korea; 7 College of Pharmacy, Chonbuk University, Jeonju, Jeonbuk 54896, Republic of Korea; 8 School of Pharmacy, Sungkyunkwan University, Suwon 16419, Republic of Korea

**Keywords:** Sirtuin 5, Lactate dehydrogenase A, Lysine succinylation, PCa progression

## Abstract

Prostate cancer (PCa) is the most commonly diagnosed genital cancer in men worldwide. Around 80% of the patients who developed advanced PCa suffered from bone metastasis, with a sharp drop in the survival rate. Despite great efforts, the detailed mechanisms underlying castration-resistant PCa (CRPC) remain unclear. Sirtuin 5 (**SIRT5**), an NAD^+^-dependent desuccinylase, is hypothesized to be a key regulator of various cancers. However, compared to other SIRTs, the role of SIRT5 in cancer has not been extensively studied. Here, we revealed significantly decreased SIRT5 levels in aggressive PCa cells relative to the PCa stages. The correlation between the decrease in the SIRT5 level and the patient’s reduced survival rate was also confirmed. Using quantitative global succinylome analysis, we characterized a significant increase in the succinylation at lysine 118 (K118su) of **lactate dehydrogenase A** (LDHA), which plays a role in increasing LDH activity. As a substrate of SIRT5, LDHA-K118su significantly increased the migration and invasion of PCa cells and LDH activity in PCa patients. This study reveals the reduction of SIRT5 protein expression and LDHA-K118su as a novel mechanism involved in PCa progression, which could serve as a new target to prevent CPRC progression for PCa treatment.

## Introduction

Prostate cancer (PCa) is the most common genital cancer in men, with about 248,530 new cases of PCa in 2021, as estimated by the American Cancer Society [1]. PCa was initially considered a cancer of elderly men, but PCa incidence in patients below age 55 is currently increasing by more than 10% in the United States [2]. Although the 5-year survival rate of localized PCa is > 99%, the outlook is relatively poor once PCa advances [3], [4]. Half of the men with castration-resistant PCa (CRPC) developed bone metastasis within two years of CRPC diagnosis [5]. PCa can spread to a variety of tissues, especially the lymph nodes and bone [6]. A previous study has shown that PCa first metastasized to the bone and then to other organs [7]. The progression from the bone to other metastatic sites is associated with decreased survival in CRPC.

A diversity of covalent post-translational modifications (PTMs) are implicated in various biological mechanisms during cancer progression [8]. Lysine residues have various types of acylation, such as acetylation, succinylation, malonylation, and glutarylation. These acylations are regulated by silent information regulator 2-like protein (sirtuin; SIRT) which removes the modified acyl groups from proteins as deacylase. SIRTs are highly conserved proteins with NAD^+^-dependent deacylase activity and are classified as class III histone deacetylases [9], [10]. There are seven SIRTs (SIRT1–SIRT7) in mammals, all of which show diverse subcellular localizations and distinct functions [11]. Numerous studies have revealed that SIRTs play critical roles in cancer progression and metastasis by regulating angiogenesis, inflammation, and epithelial-to-mesenchymal transition (EMT) [12], [13], [14].

In this study, we tried to explore the regulatory mechanisms involved in PCa secondary metastasis from bone to other organs using two PCa cell lines, PC-3 and PC-3M [15]. The PC-3 cell line is commonly used to study PCa as the leading cause of death in PCa patients is bone metastasis [6], [16]. The PC-3M cell line, which is a highly metastatic subline of PC-3 isolated from a PC-3-induced mouse tumor and shows increased migration compared to PC-3 cells, is used as a positive control [15].

We examined SIRT expression in these two PCa cell lines and found that SIRT5 expression was significantly decreased with PCa progression. SIRT5 is known as the deacylase of several acylations but plays the most important role in desuccinylation [11], [17]. Various roles of lysine succinylation (Ksu) in individual proteins have been reported, particularly in relation to cancer progression and metastasis [18], [19], [20]. For example, S100A10 was found to be succinylated at lysine 47 in gastric cancer. SIRT5 regulates desuccinylation, and the levels of succinylated S100A10 were increased in human gastric cancer [21]. Thus, in this study, we identified the specific protein substrate of SIRT5. Based on a comparative global succinylome study in PC-3 and PC-3M cell lines, we proposed the role of Ksu of the identified protein substrate in increasing the metastatic potential of PCa.

## Results

### SIRT5 expression is significantly down-regulated in advanced PCa

**Figure 1:**
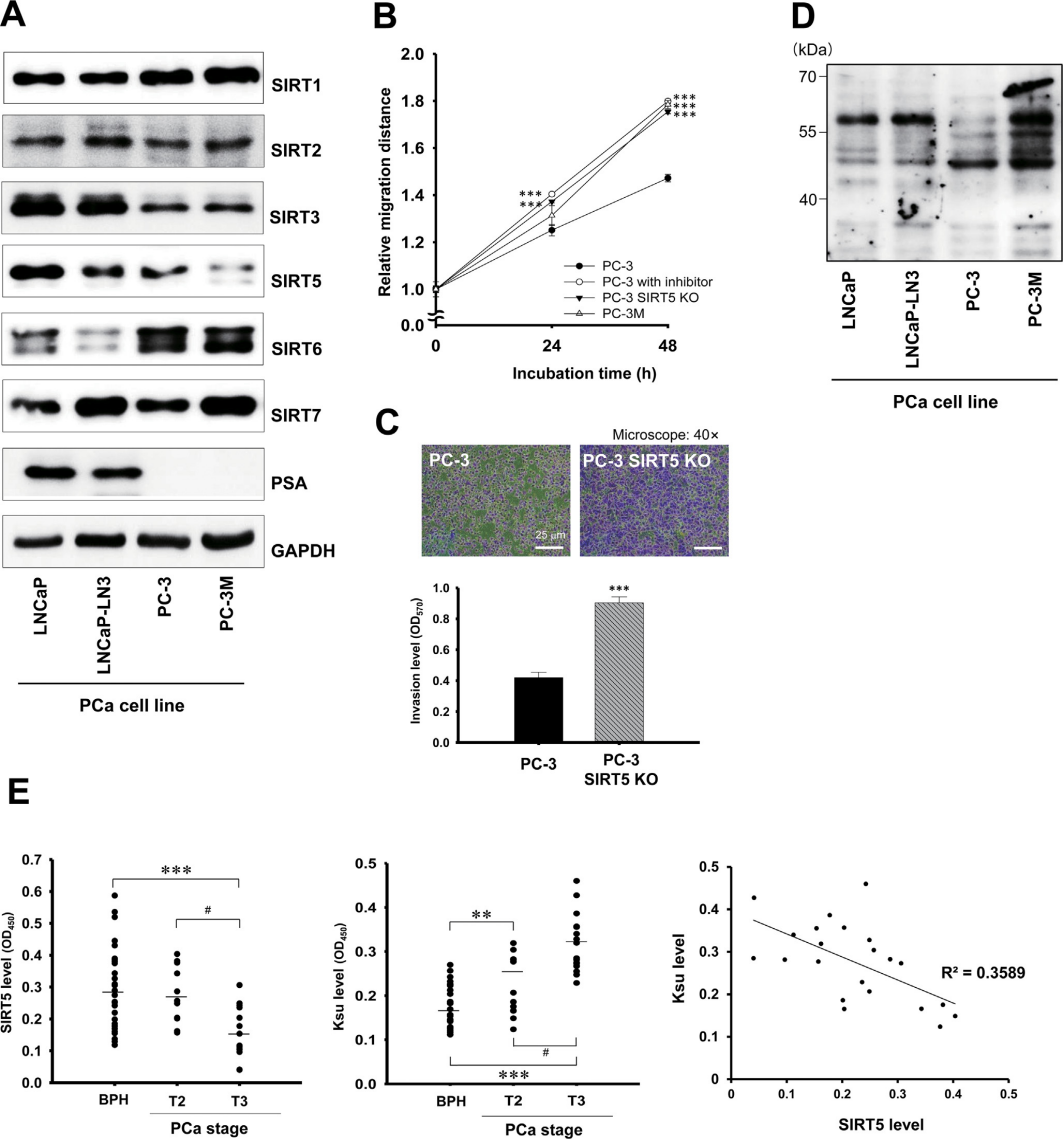
**Characterization of SIRT5 in PCa** **A.** The protein expression level of SIRT5 was significantly decreased in PC-3M compared to PC-3 cells. Levels of six SIRT members were analyzed by Western blotting with indicated antibodies in PCa cell lines. GAPDH served as a loading control. **B.** SIRT5 regulates cell migration. Cell migration was evaluated by a wound-healing assay in PC-3 cells after treatment with an SIRT5 inhibitor (HY-112634) and in PC-3 *SIRT5*-KO and PC-3M cells (*n* = 3). **C.***SIRT5* knockout (KO) promoted cell invasion. Cells that invaded through matrigel were stained with crystal violet (*n* = 3). Representative images depict invaded cells (40×). Scale bars, 25 μm. **D.** The global Ksu levels increased in the PC-3M cells. Levels of Ksu were analyzed using Western blotting with anti-pan-Ksu antibody in whole lysate-derived PCa cells. **E.** SIRT5 protein expression was significantly ‘decreased depending on the stage of PCa. In contrast, Ksu level was significantly increased depending on the stage of PCa. The correlation between SIRT5 levels and Ksu levels in PCa tissues was determined in the scatter plot. Levels of SIRT5 protein and Ksu were confirmed based on sandwich ELISA in BPH, T2, and T3 stage PCa tissues with indicated antibodies. Data are presented as mean ± SE. Statistical analysis was performed using one-way analysis of variance and presented as follows: **, *P* < 0.01; ***, *P* < 0.001 vs. BPH; #, *P* < 0.01 *vs.* T2. See also Figures S1 and S2. PCa, prostate cancer; SIRT, sirtuin; Ksu, lysine succinylation; BPH, benign prostatic hyperplasia; OD, optical density.

**Supplementary Figure S1:**
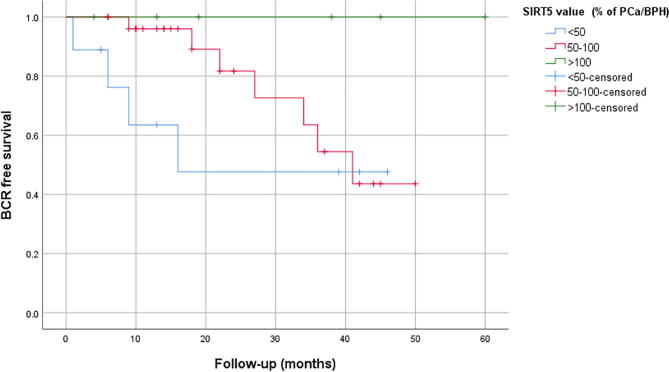


To identify the specific SIRT isoform related to PCa progression, we examined the expression level of SIRT family proteins (SIRT1 through SIRT7, except SIRT4) in four representative PCa cell lines, including androgen-dependent LNCaP and LNCaP-LN3 cells and androgen-independent PC-3 and PC-3M cells (Figure 1A). Protein expression of SIRT1 and SIRT7 was up-regulated in PC-3M cells, which is consistent with previous studies [26], [27]. Meanwhile, protein expression of SIRT5 decreased with PCa progression from LNCaP to PC-3M cells. Protein expression of SIRT5 was decreased in the progressed cells (LNCaP-LN3) compared to LNCaP cells and was dramtically down-regulated in PC-3M cells, which are more progressive than PC-3 cells. Given PC-3M cells exhibit enhanced metastasis compared to PC-3 cells, decreased SIRT5 expression in PC-3M cells may be associated with metastasis [15]. SIRT5 is a cytosol localized protein, primarily present in the mitochondria [11]. In the present study, we confirmed SIRT5 distribution by confocal imaging and immunoblotting analyses after subcellular fractionation (Figure S1A and B). Protein expression of SIRT5 was markedly down-regulated in the cytosol and mitochondria of PC-3M cells compared to PC-3 cells. No significant difference in *Sirt5* mRNA expression was detected between these two cell lines (Figure S1C). As PC-3M is a cell line isolated from PC-3, there is no genetic difference regarding SIRT5 expression. It can be assumed that the difference in the protein expression of SIRT5 might be caused by factors other than gene expression. In contrast to the decrease in the protein expression of SIRT5, protein expression of SIRT2 was increased in aggressive cancer cells (Figure 1A). Our findings in the aggressive PCa cell line are in good accordance with previous reports that SIRT2 promotes migration and invasion in various cancers [22], [23].

### SIRT5 significantly reduces cell migration and invasion in PCa

To test the role of SIRT5 in PCa cells, we investigated whether SIRT5 affects the migration and invasion of PCa cells. Migration assays were performed after the cells were treated with a selective peptide inhibitor of SIRT5, to determine whether SIRT5 regulates the migratory ability of PCa cells [24], [25] (Figure 1B; Figure S1D). The results showed that migration ability of PCa cells was increased when SIRT5 activity of PCa cells was inhibited. Next, the clustered regularly interspaced short palindromic repeats (CRISPR)/CRISPR-associated protein-9 (Cas9) endonuclease genome editing system was used to knock out the *SIRT5* gene in PC-3 cells (termed PC-3 *SIRT5*-KO). Western blotting analysis showed that SIRT5 levels were markedly decreased, but no changes in the levels of other SIRT proteins were observed in PC-3 *SIRT5*-KO cells (Figure S1E), indicating the specificity of *SIRT5* knockout. To evaluate whether SIRT5 affects cellular growth rate, we used the cell counting kit-8 (CCK-8) assay [26], [27] to compare proliferation of normal (PC-3), control (vehicle), PC-3 *SIRT5*-KO, and PC-3M (positive control) cells (Figure S1F). We found that *SIRT5*-KO cells proliferated significantly faster than normal PC-3 cells (*P* < 0.001) indicating that SIRT5 can regulate the proliferation of PC-3 cells. Next, a migration assay [27], [28] was performed in the aforementioned cells to assess the effect of SIRT5 on their migratory ability. Interestingly, *SIRT5*-KO cells migrated significantly faster than PC-3 cells as measured by TScratch software (*P* < 0.001), and increased in migration was also observed in PC-3 cells treated with SIRT5 inhibitors (Figure 1B; Figure S1D), suggesting that SIRT5 regulates the migration of PCa cells. Furthermore, invasion assays [29] were performed to evaluate the invasiveness of PCa cells upon *SIRT5*-KO (Figure 1C). The results demonstrated an increased invasion of PC-3 *SIRT5*-KO cells through the matrigel to the bottom membrane. These two cell lines were eluted with methanol with absorbence measured at 570 nm [29]. Invasion assay data for PC-3 and PC-3 *SIRT5*-KO cells demonstrated that SIRT5 depletion in PC-3 cells is associated with increased invasiveness. Taken together, these results indicate that SIRT5 is an important factor in increasing cell migration related to PCa progression.

### Decreased SIRT5 is associated with PCa progression

**Supplementary Figure S2:**
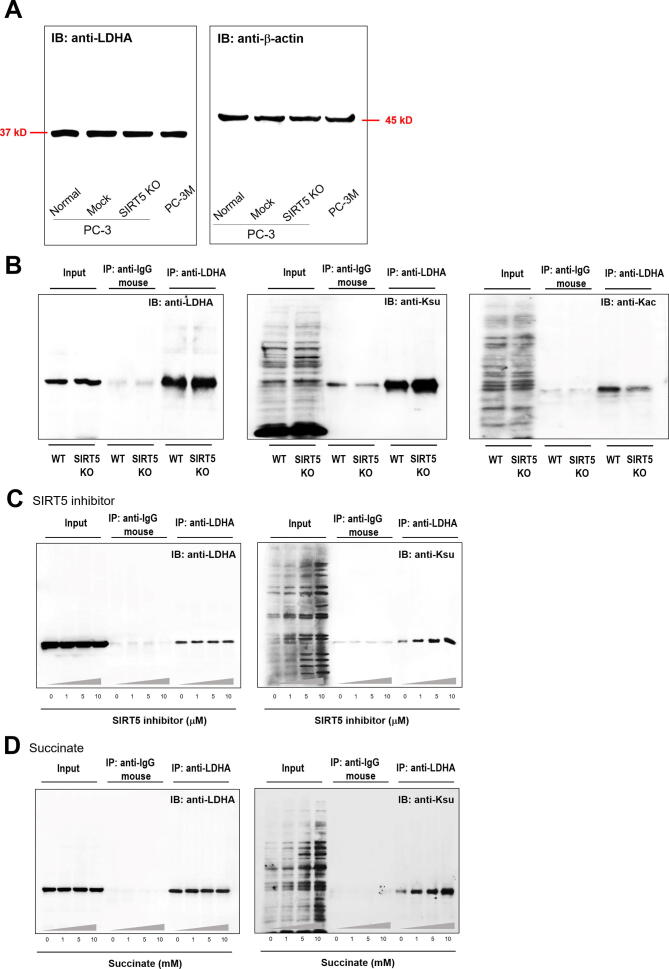


In Figure 1A, protein level of SIRT5, a representative desuccinylase, was decreased in PC-3M cells. Consequently, an increase in the Ksu level was observed in these cells as well (Figure 1D). The increase in Ksu level in PC-3M cells (compared to other cells) is more obvious than that of other acylations, including K-acetylation, K-b-hydroxybutylation, K-malonylation, and K-glutarylation in (Figure S1G). Furthermore, low levels of SIRT5 were significantly correlated with T grade increase (T2 to T3) (*P* = 0.03) but less strongly associated with age, weight, body mass index, and Gleason score (Table S1). To verify the correlation between SIRT5 expression level and PCa progression, an enzyme-linked immunosorbent assay (ELISA) was carried out to assess the levels of SIRT5 and Ksu in PCa tissues [benign prostatic hyperplasia (BPH) as control, T2 and T3 stages; Figure 1E]. We found that SIRT5 levels were significantly decreased in more advanced PCa tissues (*P* < 0.05), which is consistent with the downregulation of SIRT5 in PC-3M cells. In contrast to the decreased SIRT5 level in PCa tissues, the Ksu level in tissue samples from patients with T3 tumor stage was significantly increased compared to those from patients with T2 tumor stage (*P* < 0.01), as a result of the reduced desuccinylation activity. Moreover, the SIRT5 protein level was negatively correlated with the Ksu levels in tissue samples from patients (Figure 1E). Patients with a lower SIRT5 level appeared to have a worse overall survival (Figure S2). The difference were not significant, possibly due to the lack of sufficient observation time and number of patients, considering the long progression of PCa.

### Succinylated proteins are identified by a global succinylome analysis

**Figure 2:**
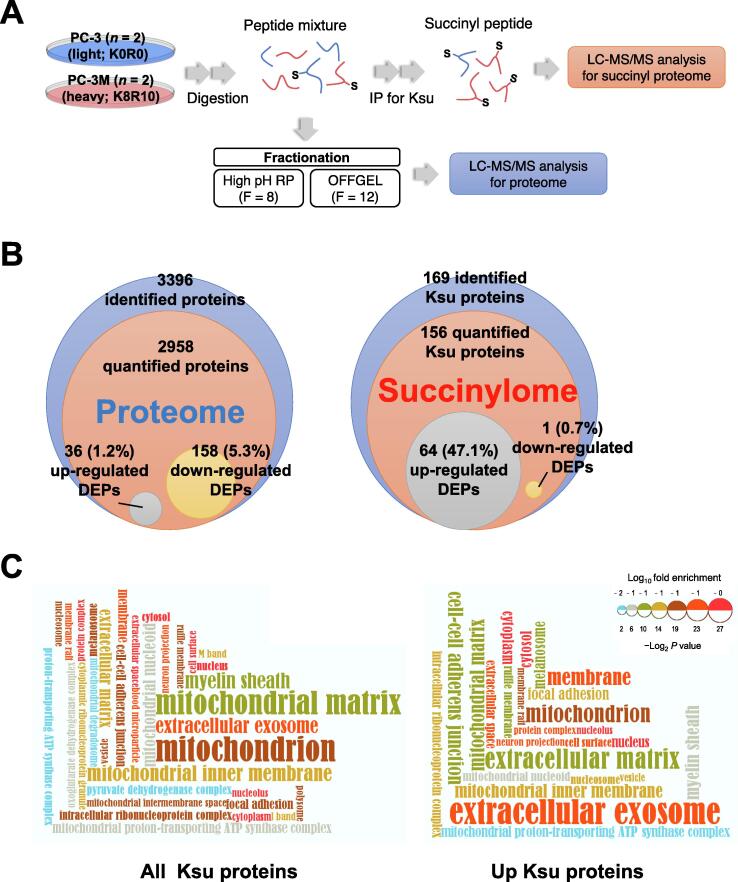
I**ncreased Ksu in the cytoplasm in PCa** **A.** Change in Ksu between PC-3 and PC-3M cells was identified by the global lysine succinylome technique. PC-3 and PC-3M cells were labeled with light or heavy amino acids in SILAC media, respectively. The peptide mixtures were enriched by IP using anti-succinyl-lysine antibody-conjugated agarose beads (*n* = 2). The succinyl-lysine proteins were identified and quantified by high-resolution tandem MS (MS/MS) analysis and peak alignment. **B.** The level of succinylation is changed more than that of proteins. Only 6.5% of the total proteins was DEPs, but 41.7% of succinylated proteins was DEPs. **C.** The succinylation level of the cytoplasm and extracellular components is hiher than those of mitochondrial compontents. All Ksu and up-regulated Ksu proteins in PC-3M cells were visualized by functional enrichment of the GOCC category. See also Figure S3 and Tables S2–S4. IP, immunoprecipitation; DEP, differentially expressed protein; GOCC, gene ontology cell component.

**Supplementary Figure S3:**
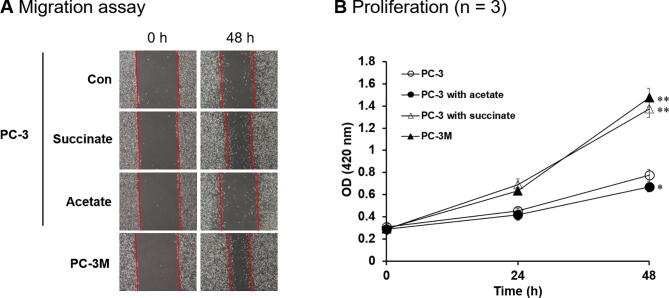


Based on the aforementioned results, it is hypothesized that the decrease in SIRT5 level could promote PCa progression. To identify the substrate of SIRT5 associated with aggressive PCa, a global protein succinylation assay was performed in PCa cell lines using stable isotope labeling by amino acids in cell culture (SILAC)-based quantitative proteomics and global immunoprecipitation (IP) enrichment [30] (Figure 2A). PC-3 and PC-3M cells were labeled with light (K0R0) and heavy (K8R10) SILAC medium, respectively. Biological replicates of two different samples were also prepared to minimize the impact of biological variation. The Pearson correlation coefficients of heavy/light labeling ratio between the replicates were 0.89 and 0.71 for proteins and Ksu peptides, respectively (Figure S3A). Ksu analysis showed that 81 of 169 (47.9%) Ksu proteins were overlapping, and 167 of the 488 (37.3%) Ksu sites were duplicated (Table S2).

We quantified 2958 proteins and in total 194 (6.5%) of them were differentially expressed proteins (DEPs), which include 36 (1.2%) up-regulated and 158 (5.3%) down-regulated proteins in PC-3M cells compared to PC-3 cells (Figure 2B; Table S2). Based on the succinylome data, 448 Ksu sites on 169 proteins were identified, and 406 of these Ksu sites in 136 proteins were quantified and normalized to protein levels (Table S3). Interestingly, 144 of 145 differentially expressed sites of Ksu were found to be increased in PC-3M cells compared to PC-3 cells. This accounted for 35.5% of the total quantified Ksu sites and 47.1% of the quantified Ksu proteins (Figure 2B). The difference between these two cell lines at the protein level is marginal as only 6.5% of the proteins quantified are DEPs, whereas the difference between these two cell lines at the Ksu level is remarkable, as 41.6% succinylated proteins were DEPs. Therefore, it is suggested that protein succinylation may be a more important factor for the aggressiveness of PCa cells. Gene Ontology (GO) enrichment analysis was conducted using DAVID [31] to compare the biological properties of total proteins containing Ksu (Ksu protein) and up-regulated Ksu proteins (Figure S3B). DAVID analysis indicated that different categories were enriched between identified total Ksu proteins and up-regulated Ksu proteins in the GO biological process (GOBP), GO cell component (GOCC), and GO molecular function (GOMF). Furthermore, GOCC analysis showed that the subcellular distribution of total Ksu proteins and that of the up-regulated Ksu proteins was different (Figure 2C). Intriguingly, total Ksu proteins appeared to be more abundant in the mitochondria (42.9%) than in the cytoplasm or nucleus. In contrast, Ksu proteins up-regulated in PC-3M cells were more dominant in the cytoplasm (46.3%). These data suggest that the substrate of SIRT5 that affects PCa progression is likely a cytosol protein.

According to the aforementioned succinylome analysis in advanced PCa, Ksu proteins up-regulated in PC-3M cells were dominated by extracellular and cytoplasmic proteins but not mitochondrial proteins (Figure 2C). Therefore, among the extracellular exosome and extracellular matrix proteins in the GOCC category, 67 up-regulated Ksu sites on 26 proteins, excluding mitochondrial, mitochondrial matrix, and mitochondrial inner membrane proteins, were selected. Furthermore, 36 increased Ksu sites in 15 cytoplasmic proteins were selected based on subcellular localization (Figure S3C). Finally, 33 up-regulated Ksu sites on 12 proteins, characteristically extracellular or cytoplasmic while excluding the mitochondrion, were selected as candidate substrates for aggressive PCa (Figure S3D; Table S4). Of the 12 proteins, lactate dehydrogenase A (LDHA) was selected because four of the seven sites capable of succinylation were indeed succinylated, and LDHA is known as a key player related to carcinogenesis according to previous studies [32], [33].

### LDHA-K118 succinylation increases LDH activity

**Figure 3:**
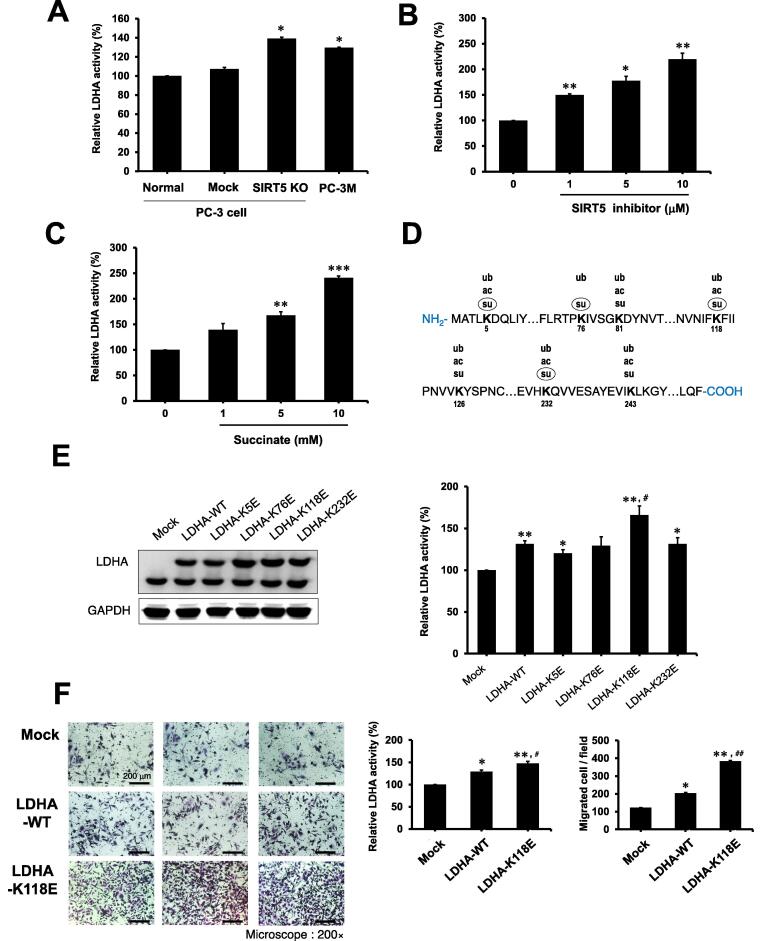
**LDHA desuccinylation by SIRT5 is related to aggressive PCa** **A.** LDHA activity was increased in SIRT5-depleted PCa in PC-3M, and PC-3 SIRT5-KO cells. LDHA activity was determined in PC-3 (Normal), Mock treated PC-3 (Mock), PC-3 SIRT5-KO (SIRT5 KO), and PC-3M cells. **B.** SIRT5 inhibition leaded to increased by LDHA activity. LDHA activity was determined by the treatment with the SIRT5 selective inhibitor (HY-112634) in PC-3 cells. **C.** Inducing succinylation increased LDHA activity. Succinate was administered to PC-3 cells to increase the succinylation level, and LDHA activity was determined. **D.** Detected succinylation sites (su) in LDHA. Among the detected sites, sites with succinylation levels increased more than two times in PC-3M are marked with circles. The acetylation (ac) and ubiquitylation (ub) sites known in phosphostie.org are indicated. **E.** LDHA with lysine (K) mutated to glutamic acid (E) at K118 residue increased LDHA activity. The four lysine residues K5, K76, K118, and K232, with up-regulated succinylation in PC-3M, were mutated to E to mimic the negatively charged succinyl-lysine modification. After PC-3 cells were transfected with LDHA-mutants using lipofectamine 3000, LDHA activities were determined. GAPDH served as a loading control (*n* = 3). **F.** The mutation from K to E of LDHA at K118 promotes cell migration and invasion. Cell migration and invasion of PC-3 cells expressed LDHA-K118E were analyzed by wound-healing and transwell invasion assays (*n* = 3). Representative images depict invading cells (200 ×). Cells that invaded through matrigel were stained with crystal violet. Bar, 25 mm. Data are presented as mean ± SE. Statistical analysis was performed using one-way analysis of variance and presented as follows: *, *P* < 0.05; **, *P* < 0.01 vs. mock; ^#^, *P* < 0.05; ^##^, *P* < 0.01 *vs.* LDHA-WT. See also Figures S5 and S6. LDHA, lactate dehydrogenase A; WT, wild type.

The high migration and invasion of PC-3M cells may be related to succinylation of LDHA as a substrate of SIRT5. LDHA activity was significantly increased in *SIRT5*-KO and PC-3M cells compared to PC-3 cells (*P* < 0.05) (Figure 3A). However, there was no change in LDHA expression in the four cell groups examined (Figure S4A). LDHA expression in wild-type (WT) and *SIRT5*-KO PC-3 cells was not changed, but succinylation was increased in *SIRT5*-KO cells (Figure S4B). Moreover, The level of lysine acetylation (Kac), which promotes LDHA degradation, was reduced in *SIRT5*-KO cells (Figure S4B) [24]. LDHA activity was also increased in a concentration-dependent manner, even when PC-3 cells were treated with a SIRT5 selective inhibitor (HY-112634) (Figure 3B). Likewise, there was no change in LDHA protein expression in response to the treatment with SIRT5 inhibitor, but the succinylation level of LDHA was increased in the similar pattern as its activity increased (Figure S4C). To verify whether LDHA succinylation leads to an increased LDHA activity, succinylation of LDHA was induced by treating PC-3 cells with succinate. Succinate treatment in PC-3 cells increased the succinylation of LDHA and also significantly increased the activity of LDHA (P < 0.01) (Figure 3C, Figure S4D). Additionally, cell migration and proliferation were increased by succinate treatment (Figure S5). Collectively the increased succinylation in LDHA could promote LDHA activity, as well as cell migration and proliferation.

Next, we tried to identify the exact lysine residue where the succinylation that regulates LDHA activity occurs. Seven succinylated sites in LDHA were identified through global succinylome studies in PC-3 and PC-3M cells at K5, K76, K81, K118, K126, K232, and K243 (Figure 3D). The locations of known acetylation and ubiquitylation in LDHA were marked with succinylation [34], [35]. In PC-3M cells, seven succinylated sites of LDHA were detected, of which four sites including K5, K76, K118, and K232 increased more than twice compared to PC-3 cells. Each *mz*/*mz* spectrum was verified manually (Figure S6). Succinylation at K5 and K118 in LDHA was reported in a previous study [11]. However, there lack quantitative or functional studies of succinylation under disease situations.

We specupate that the four Ksu sites of LDHA with increased succinylation in PC-3M cells could be substrates for SIRT5. To identify the specific sites that regulate LDHA activity, each of the four succinylated sites was separately mutated to glutamic acid (E), mimicking the physiological properties of protein succinylation. Point mutations of K5E, K76E, K118E, and K232E resulted in a significant induction in LDHA succinylation (*P* < 0.05) (Figure 3E). Among the four mutations, K118E mutated LDHA showed the highest activity, indicating that LDHA activity can be increased by succinylation of LDHA at K118. It was also confirmed that invasion of cells overexpressing K118E-mutated LDHA was increased (Figure 3F). In order to verify succinylation at K118 of LDHA, succinyl-LDHA K118 antibody was newly generated by immunizing rabbits with succinyl-K118 peptide conjugated with a carrier protein. The selectivity of the generated antibody was confirmed by dot-blot assay and competitive immunoblot using synthesized LDHA succinyl-K118 peptide (Figure S7).

### LDHA-K118 succinylation is the key regulator in advanced PCa

**Figure 4:**
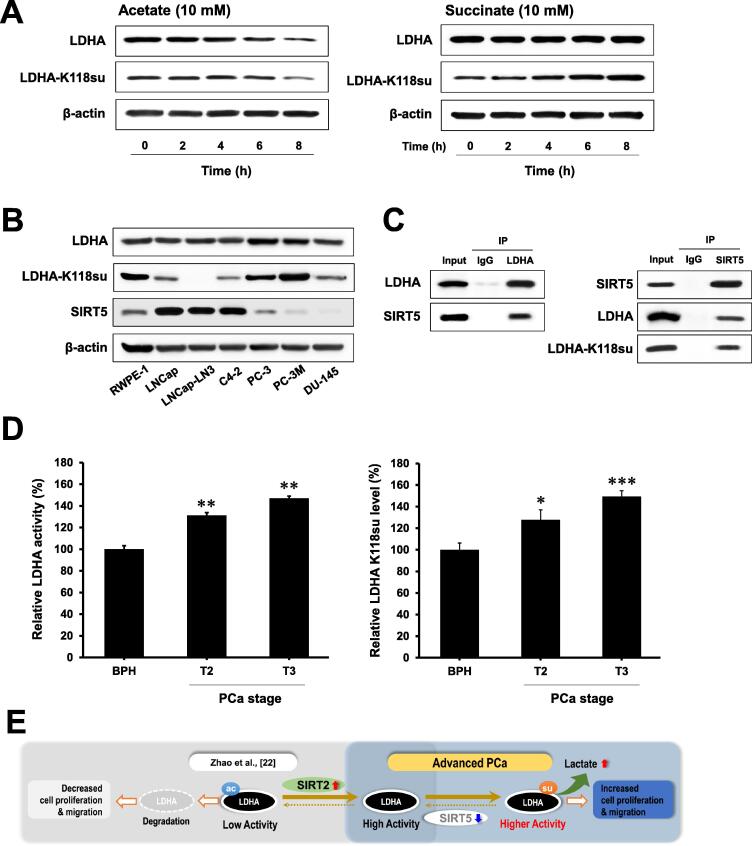
**LDHA succinylation promotes PCa progression** **A.** LDHA acetylation induces LDHA degradation. Succinate increased succinylation at the K118 residue of LDHA. The levels of LDHA and LDHA-K118su were determined by Western blotting after treatment with acetate or succinate in PC-3 cells for 8 h. β-actin served as a loading control. **B.** The protein expression level of LDHA, LDHA-K118su, and SIRT5 in seven types of PCa cell lines was evaluated by immunoblot. β-actin served as a loading control. **C.** SIRT5 interacts with LDHA. The immunoblots showed the IP of SIRT5 with anti-LDHA antibodies in PC-3 cells and IP of LDHA with anti-SIRT5 antibodies in PC-3 cells, respectively. **D.** As PCa progresses, LDHA is succinylated at K118 becomes the K118su residue, along with increased LDHA activity. LDHA activity was determined in PCa tissues classified by pathological grade (BPH, T2, and T3 grades). The K118su level of LDHA in PCa tissue was measured by ELISA using the LDHA-K118su antibody. **E.** Working model of LDHA succinylation at K118 in aggressive PCa cells. LDHA degradation is regulated according to acetylation at the K5 position by SIRT2, according to Zhao et al. 2013 [24]. In PCa, the SIRT5 protein level is reduced; The succinylation at K118 of LDHA as the substrate of SIRT5 is maintained. Succinylated LDHA is more active than the native LDHA, which can increase lactate production, ultimately leading to PCa progression. Data are presented as mean ± SE. Statistical analysis was performed using one-way analysis of variance and presented as follows: *, *P* < 0.05; **, *P* < 0.01; ***, *P* < 0.001 *vs*. BPH. K118su, K118 succinylation.

As reported in previous studies, LDHA activity is regulated by protein modification, such as acetylation at K5 or phosphorylation at Y10 [22], [36]. K5 acetylation of LDHA is known to decrease LDHA level by inducing lysosomal degradation of LDHA in human pancreatic cancer [24]. Acetylation and succinylation of LDHA may play opposite roles in the regulation of LDHA level in cancer. To evaluate the correlation between acetylation and succinylation in LDHA, PC-3 cells were treated with acetate and succinate, respectively. When acetate was added to induce LDHA acetylation, the protein level of LDHA was reduced as reported in the previous study [24] (Figure 4A). PC-3 cells treated with succinate showed increased migration and proliferation, which are comparable to that of PC-3M cells, whereas acetate treatment did not increase migration and proliferation of PC-3 cells, compared to the untreated PC-3 cells (Figure S5). When cells were treated with succinate, succinylation levels at the K118 residue was increased with time (Figure 4A). Succinylation at LDHA-K118 tended to decrease with increased SIRT5 expression in several types of PCa-related cell lines (Figure 4B).

To verify the possibile of interaction between SIRT5 and LDHA, Co-Immunoprecipitation (Co-IP) experiments were performed in PC-3 cells using anti-SIRT5 and anti-LDHA, respectively. In the Co-IP experiment, when LDHA and SIRT5 antibodies were used, co-precipitation of SIRT5 and LDHA was demonstrated using immunoblot, respectively (Figure 4C). Conversely, when SIRT5 was immunoprecipitated, LDHA was precipitated together. Based on these data, we speculate that LDHA-K118 succinylation (LDHA-K118su) is desuccinylated by SIRT5. In patient tissues, as PCa progressed, LDHA activity increased significantly in tissue samples from the T2 and T3 stages, compared to BPH (*P* < 0.01) (Figure 4D), which was associated with SIRT5 reduction (Figure 1E). In Figures 1E, we showed that the levels of total Ksu were increased along with PCa progression. In line with this obsefrvation but more accurately, using the LDHA K118su-specific antibody that we generated, we found that the level of LDHA-K118su in PCa tissues was also significantly increased according to the stage of PCa progression (*P* < 0.05) (Figure 4D). At advanced PCa stages, the decrease in the level of SIRT5 would result in an increase in he level of succinylated LDHA, which is more active than intact LDHA. We thus propose that succinylated LDHA promotes PCa progression (Figure 4E).

## Discussion

SIRT5 is involved in cell metabolism, including glycolysis, tricarboxylic acid cycle, fatty acid oxidation, nitrogen metabolism, pentose phosphate pathway, antioxidant defense, and apoptosis [37]. Although SIRT5 plays important regulatory roles in tumor progression in the liver and gastric cancer, the role of SIRT5 in cancer has not been extensively studied compared to other SIRTs [38], [39]. Recent studies have shown that SIRT5 is associated with cancer progression through the regulation of succinylation of specific proteins involved in cancer progression [21], [40], [41]. Here, SIRT5 levels in PC-3M cells were significantly lower than those in PC-3 cells as well as other androgen-dependent PCa cell lines (LNCaP and LNCaP-LN3). SIRT5 expression and overall survival ratio showed a clear correlation, although not statistically significant. SIRT5 expression was significantly decreased as PCa progressed to T2 and T3 stages, whereas K118su and LDHA activity were increased. Therefore, we speculate that the increased succinylation of LDHA resulting from the decreased SIRT5 protein expression regulates the aggression of PCa by increasing LDHA activity.

To investigate the molecular mechanism of reduced SIRT5 protein expression in PC-3M cells, we measured gene expression of *SIRT5* and no remarkable changes were detected between PC-3 and PC-3M cells (Figure S1C). In addition, *Sirt5* expression was not affected by treatment with a protein synthesis inhibitor (cycloheximide), while treatment with a proteasomal inhibitor (MG132) or a lysosomal inhibitor (chloroquine) did not alter SIRT5 expression either (data not shown). These results indicate that the decrease in SIRT5 in PC-3M cells is not regulated by transcriptional regulation or protein stability. Recently, *SIRT5* was reported to be a downstream target of several microRNAs, including miR-299-3p, miR-19b, and miR-3677-3p, which regulate the proliferation, migration, and invasion of cancer cells [42], [43], [44]. However, further studies are required to understand and determine the microRNAs related to SIRT5 decrease in PC-3M cells.

LDHA shows a high expression profile and activated status in many cancers [32]. A number of previous studies have shown a correlation between LDHA activity and tumor growth and metastasis [33], [45], [46], [47], [48]. LDHA plays an important role in metastasis and hepatocellular carcinoma cell growth [45]. LDHA expression was up-regulated in breast cancer and correlated with poor clinical outcomes of breast cancer [46]. When LDHA expression was reduced by small interfering RNA, the progression of lymphoma growth was inhibited [47]. Especially in PCa, high LDHA expression in PC-3 cells induces a favorable tumor microenvironment for tumor progression [48]. As a result, increased LDHA activity promotes tumor progression.

The correlation between increased LDHA expression and cancer progression suggests that LDH activity is an important factor to consider for cancer treatment. However, there are no suitable LDHA inhibitors for therapertic use in the clinic yet [33]. The regulation of LDH activity in cancer cells is very important for cancer progression, and protein modification plays a key role in regulating LDH activity. As mentioned previously, LDH activity is regulated by protein modification, such as acetylation at K5 or phosphorylation at Y10 [22], [36]. Acetylation at K5 of LDHA in human pancreatic cancer increases the lysosomal degradation of LDHA, and LDH activity was inhibited [24]. In breast and colorectal cancer stem cells, LDHA phosphorylation at Y10 was positively correlated with the progression of metastasis and cell invasion [36], [49].

In this study, we reported a new mechanism for regulating LDHA activity in aggressive PCa, that is, activity of LDHA is increased by K118su and SIRT5 regulates the desuccinylation of LDHA. Overall, SIRT5 increases protein stability and delay degradation of LDHA due to succinylation, resulting in its increased activity. The mechanism could be explained by the increased lactate levels in cancer cells resulting from the increased LDHA activity, which promotes cancer cell growth [50], [51], [52]. Even in PCa cells, decreasing the lactate concentration significantly inhibited the growth of cancer cells [52]. Regarding LDHA activity, K118su of LDHA after a decrease in SIRT5 may be related to ubiquitination modified at the same site (Figure 3D) [34]. It is speculated that LDHA activity is maintained by inhibiting protein degradation because K118su does not proceed with ubiquitylation. Since succinylated LDHA shows higher activity than intact LDHA, a lower protein level of SIRT5 could imply an increase in the highly active form of LDHA (Figure 4E). A previous study reports that there is no change in LDHA activity in the *SIRT5*-KO cells of colorectal cancer [41]. Although the level of succinylated-LDHA was not examined in that study, it could be assumed that succinylation of LDHA is also a key process like desuccinylation. Since the succinylation levels of LDHA did not increase in colorectal cancer, it is speculated that the expression of SIRT5 would not affect the change in LDHA activity. Both desuccinylation by SIRT5 and succinylation by succinyl-transferase should be considered for the potential role of LDHA succinylation as a key regulator in promoting cancer progression.

## Conclusion

PCa is the most common type of genital cancer in men, accounting for the highest number of all new cancer cases, and is the third leading cause of cancer-related deaths in the world. SIRT5 protein expression was significantly down-regulated in advanced PCa based on studies using PCa cell lines, and reduced SIRT5 expression was associated with a decreased survival rate in PCa patients. SIRT5, a representative desuccinylase, regulates protein succinylation at the PCa stage. Through global succinylome analysis, we identified that LDHA-K118su is regulated by SIRT5, which increased LDHA activity. As the substrate site of SIRT5, LDHA-K118su significantly increased as PCa progressed and showed a positive correlation with increased migration and invasion of PCa cells. Therefore, LDHA activity, which promote PCa progression, is up-regulated by an increased succinylation at K118 and LDHA is a substrate for SIRT5, suggesting a new mechanism to inhibit PCa progression. In further studies, it is necessary to evaluate whether the succinylation of LDHA at K118 could be a target for the development of novel antitumor drugs.

## Materials and methods

### Clinical samples of prostate tissues

Normal tissues (BPH) and PCa tissues (separated into T2 and T3) were stored at −80 °C before use.

### PCa cell line culture

PCa cell lines, including LNCaP, LNCaP-LN3, PC-3, and PC-3M, were purchased from the Korea Cell Line Bank (Seoul, Korea). The detailed experimental methods are described in File S1.

### Western blotting analysis

PCa cell lines were lysed using radioimmunoprecipitation assay buffer (Catalog No. 89900, Thermo Fisher Scientific, Waltham, MA). Protein concentrations were measured by BCA assay (Catalog No. 23224, Thermo Fisher Scientific). Samples were separated via 10% sodium dodecyl sulfate (SDS)-polyacrylamide gel electrophoresis (PAGE) for 90 min and transferred to polyvinylidene fluoride blotting membranes (0.2 μm pore size; GE Healthcare, Chalfont St. Giles, UK) for 2 h. The membrane was blocked in 5% bovine serum albumin for 2 h. Primary antibodies against SIRT1 (Catalog No. 8469S, Cell Signaling Technology Technology, Beverly, MA), SIRT2 (Catalog No. 12672S, Cell Signaling Technology), SIRT3 (Catalog No. 5490S, Cell Signaling Technology), SIRT4 (Catalog No. ab231137, Abcam, Cambridge, UK), SIRT5 (Catalog No. 8782S, Cell Signaling Technology), SIRT6 (Catalog No. 12486S, Cell Signaling Technology), SIRT7 (Catalog No. 5360S, Cell Signaling Technology) and succinyl-lysine (PTM Biolabs, Chicago, IL) were used to capture SIRT1-SIRT7 proteins overnight at 4 °C. After incubation with horseradish peroxidase (HRP)-conjugated secondary antibody for 1 h, the membranes were washed in Tris-buffered saline-0.1% Tween 20 (TBS-T) three times for 15 min. ECL prime Western blotting detection reagent (RPN2232, Citiva) was used to visualize the blots.

### Wound-healing and transwell invasion assays

A wound-healing assay was performed to observe the migratory capacity of PC-3 and *SIRT5*-KO cells. Cells (0.4 × 10^6^ per well) were seeded in a 6-well plate (Corning, Corning, NY) and cultured to ∼ 90% confluence. A sterilized 1000 μl tip was used to scratch the cell monolayer artificially, and debris was then removed by washing with phosphate-buffered saline (PBS). Next, wounded cells were observed under a microscope at 12 h intervals and imaged with a Leica Las EZ microscope (Olympus, Tokyo, Japan).

To evaluate the invasiveness of PC-3 and PC-3 *SIRT5*-KO cells, a transwell invasion assay was performed with 200 μg/ml matrigel matrix (Corning) and 8 μm pore-permeable supports (Corning). Cells were resuspended in serum-free medium and seeded (5 × 10^4^) in the upper chamber, and the medium containing 10% FBS was added into the lower chamber. After 24 h, a cotton swab was used to remove the non-invading cells in the upper chamber. Cells that migrated through the matrigel matrix were fixed with 4% paraformaldehyde for 10 min and stained with 0.5% crystal violet (Catalog No. 61135, Sigma-Aldrich, St. Louis, MO) for microscopy. Finally, cells attached to the membrane were added. Optical density was measured at 570 nm.

### Construction of *SIRT5*-KO cell lines using the CRISPR/Cas9 gene-editing system

We used the CRISPR/Cas9 system to knock out SIRT5. PC-3 cells were transfected with either the control or *Sirt5* CRISPR/Cas9 plasmids according to the manufacturer’s instructions (Sigma-Aldrich). The transfection efficiency was determined by a green fluorescent protein (GFP) signal. After 24 h, GFP-expressing PC-3 cells were sorted by FACS Aria III (BD Life Sciences, Franklin Lakes, NJ) and maintained with a complete medium. SIRT5 depletion by the CRISPR/Cas9 *Sirt5* but not the control plasmid was confirmed by Western blotting.

### Proliferation assay

Cells (1.5 × 10^4^ per well) were cultured in a 96-well plate in a complete RPMI medium. After 24 h, proliferation was determined by CCK-8 (Catalog No. CK04, Dojindo, Tokyo, Japan). The CCK-8 solution was added to the plate and incubated for 30 min in a humidified incubator. The plate was measured at 450 nm after the appropriate color change was observed.

### Global IP for comparative succinylome analysis to enrich K118su

Anti-succinyl-lysine antibody-conjugated agarose beads (40 μl) were prewashed with 1 ml PBS followed by centrifugation at 1000 *g* at 4 °C for 1 min. To enrich lysine succinylated (K118su) peptides, 5 mg desalted peptides were dissolved in 1 ml NETN buffer [50 mM Tris-HCl (pH 8.0), 100 mM NaCl, 1 mM EDTA, and 1% NP-40] and incubated on a rotator at 4 °C for 16 h. The beads were washed three times with 1 ml NETN and twice with 1 ml ETN [50 mM Tris-HCl (pH 8.0), 100 mM NaCl, 1 mM EDTA] and 1 ml liquid chromatography/mass spectrometry (LC/MS)-grade water using centrifugation at 1000 *g* at 4 °C for 1 min in between washes. The enriched K118su peptides were eluted in 70 μl of 0.15% trifluoroacetic acid (TFA) by gentle mixing. After centrifugation at 1000 *g* for 1 min, TFA (50 μl; 0.15%) was added to the beads to improve the recovery efficiency. Finally, 120 μl of the enriched K118su peptides were dried in a speed-vacuum system and then desalted twice using a C18 zip tip. Samples were analyzed using LTQ-velos Orbitrap connected to the Eksigent nanoLC system at the mass spectrometry convergence research center, and detailed analysis methods are described in File S1.

### Site-directed mutagenesis

Human LDHA plasmid was purchased from Origene (Rochville, MD). Succinylation mutants of LDHA were generated using a site-directed mutagenesis kit (GeneAll, Seoul, Korea) by converting each lysine residue (K5, K76, K118, and K232) to glutamic acid (codon change from AAG or AAA to GAG or GAA). The mutation was confirmed by DNA sequencing conducted at Cosmogenetech (Seoul, Korea).

### Transient transfection and *in vitro* migration assay

To express exogenous proteins, PC-3 cells were mock-transfected or transfected with 0.5 to 4 μg LDHA-WT or LDHA-mutant using lipofectamine 3000 (Catalog No. L3000-008, Invitrogen, Carlsbad, CA). For the migration assay, cell migration was analyzed using transwell migration assay chambers (BD Life Sciences). PC-3 cells were transfected with 4 μg plasmid DNA and incubated for 24 h. Then, 1.5 × 10^5^ cells were transferred to transwell chambers to perform a migration assay and incubated for 24 h on transwell chambers.

### LDHA activity colorimetric assay

LDHA activity was performed as instructed by the manufacturer (Catalog No. K726-500, BioVision, Milpitas, CA). In brief, 0.1 g tissues or 1 × 10^6^ cells were homogenized in cold assay buffer and then centrifuged at 10,000 *g* at 4 °C for 15 min. The supernatants were used for assay and 50 ml of the sample was added to a 96-well plate. Then, 50 μl of the reaction mix was added. OD_450_ was measured at time 0 and then measured again after incubation at 37 °C for 30 min and then calculated according to the kit protocol.

### Development and characterization of K118-specific antibodies

The polyclonal antibody specific for succinylated LDHA at K118 (anti-succinylated K118-LDHA, Catalog No. CTM-121) was produced in collaboration with PTM biolabs (Hangzhou, China). Rabbits were immunized with succinylated LDHA peptides (CRNVNIF-Ksu-FIIPNVVK and RKRNVNIF-Ksu-FIIPNC). Antisera from immunized rabbits were first depleted with the unmodified peptide and then affinity purified using the succinylated K118 peptides.

### Statistical analysis

All experiments were repeated at least three times. Data are expressed as mean ± standard error (SE). Differences among groups were evaluated using Student’s *t*-tests, and the significance among groups was determined using a one-way analysis of variance followed by a Bonferroni’s posthoc test by IBM SPSS statistics version 21. The statistical parameters used for the specific sets of data are described in the figure legends.

## Ethical statement

The biospecimens used in this study were provided by the National Biobank of Korea-Kyungpook National University Hospital (KNUH, 2020-10-018), a member of the Korea Biobank Network. Data were obtained (with informed consent) under institutional review board-approved protocols.

## Data availability

The data have been deposited to the ProteomeXchange Consortium via the PRIDE partner repository (ProteomeXchange: PXD022005), and are publicly accessible at https://proteomecentral.proteomexchange.org.

## Competing interests

The authors have declared no competing interests.

## CRediT authorship contribution statement

**Oh Kwang Kwon:** Formal analysis, Visualization, Writing – original draft. **In Hyuk Bang:** Methodology, Visualization. **So Young Choi:** Formal analysis, Methodology, Writing – original draft. **Ju Mi Jeon:** Formal analysis. **Ann-Yae Na:** Formal analysis. **Yan Gao:** Formal analysis. **Sam Seok Cho:** Formal analysis. **Sung Hwan Ki:** Formal analysis, Resources. **Youngshik Choe:** Methodology. **Jun Nyung Lee:** Resources. **Yun-Sok Ha:** Resources. **Eun Ju Bae:** Resources. **Tae Gyun Kwon:** Resources, Conceptualization, Supervision. **Byung-Hyun Park:** Resources, Conceptualization, Writing – review & editing. **Sangkyu Lee:** Conceptualization, Writing – original draft, Writing – review & editing, Supervision. All authors have read and approved the final manuscript.

## Figures and Tables

**Supplementary Figure S4:**