# Supplementary material for: LDHA Desuccinylase Sirtuin 5 as A Novel Cancer Metastatic Stimulator in Aggressive Prostate Cancer
Source: Genomics Proteomics Bioinformatics. 2022 Mar 9;21(1):177–89. doi: 10.1016/j.gpb.2022.02.004 (PMC10372916; doi:10.1016/j.gpb.2022.02.004)
Supplement: Supplementary File S1 [file mmc1.docx]

**File S1 Supporting methods**

**Materials**

For screening of lysine acylation in prostate cancer (PCa), primary antibodies specific to pan anti-succinyl-lysine antibody (PTM-401; PTM Biolabs), pan anti-acetyl-lysine antibody (PTM-101; PTM Biolabs), pan anti-malonyl-lysine antibody (PTM-901; PTM Biolabs), pan anti-glutaryl-lysine antibody (PTM-1151; PTM Biolabs), and pan anti-3-hydroxy-butyryl-lysine antibody (PTM-1201; PTM Biolabs) were purchased. Horseradish peroxidase (HRP)-linked mouse and rabbit secondary antibodies were obtained from Cell Signaling Technology (Beverly, MA, USA).

**PCa cell-line culture**

The PCa cell lines, including LNCaP, LNCaP-LN3, PC-3, and PC-3M, were purchased from the Korean Cell Line Bank. RWPE1 as a normal prostate cell line was obtained from the American Type Culture Collection (Manassas, VA, USA). Cancer cell lines were cultured in RPMI 1640 medium supplemented with 10% fetal bovine serum (FBS) and 1% penicillin G and streptomycin as antibiotics at 37 °C in an atmosphere of 5% CO_2_ in a humidified incubator. The normal prostate cell line was grown in keratinocyte serum-free medium (SFM) (Gibco, Thermo Fisher Scientific, Bremen, Germany) with a human keratinocyte growth supplement kit (Gibco, Thermo Fisher Scientific), including human recombinant epidermal growth factor and a bovine pituitary extract.

**Sandwich ELISA**

To measure the level of SIRT5 and succinyl-lysine (Ksu) in prostate tissues, 50 μl/well of mouse monoclonal antibody for SIRT5 and Ksu diluted with 0.2 M sodium bicarbonate (2 μg/ml) was used to coat a sterilized 96-well polystyrene plate (Corning) for 2 h. Next, the plate was washed three times with Tris-buffered saline-0.1% Tween 20 (TBS-T) and incubated with 3% Bovine Serum Albumin (BSA) in TBS-T overnight at 4°C. Normal prostate tissues and PCa tissues lysed with RIPA buffer were incubated for 5 h at room temperature (RT) and then washed again. Then, 50 μl/well detection antibody (SIRT5 rabbit polyclonal antibody diluted with 3% BSA) was added for overnight incubation at 4 °C. After washing the plate three times, a secondary antibody (HRP-conjugated rabbit antibody) was added for 2 h, followed by five times of washes. Finally, the 3,3', 5,5"-tetramethylbenzidine (TMB) substrate (Thermo Fisher Scientific, Waltham, MA, USA) was added to each well to visualize the appropriate color. A stop solution (Thermo Fisher Scientific) was used to halt the color change to ensure precise measurement. The plate was measured for optical density at 450 nm.

**Immunofluorescence staining**

The cells were fixed with 3.2% paraformaldehyde (Electron Microscopy Sciences, Hatfield, PA, USA) for 30 min at RT. The cells were permeabilized with 0.1% Triton X-100 in PBS (PBST) and then blocked with 10% normal goat serum (Vector Laboratories, Burlingame, CA, USA) in PBST for 1 h at 37 °C. The cells were incubated with primary antibodies against SIRT5 (Abcam) and COX IV(Abcam) diluted in PBST containing 2% normal goat serum at 4 °C overnight and then washed thrice for 10 min. Subsequently, the cells were then incubated with Alexa 488- or 568-conjugated secondary antibodies for 3 h at RT followed by staining with 4′,6-diamidino 2-phenylindole at RT for 8 min. The samples were mounted on slides using Vectashield mounting medium and imaged using a confocal microscope (Leica Biosystems, Buffalo Grove, IL, USA) using 600× objective for imaging.

**RT-qPCR**

Total RNA was isolated from PC-3 and *SIRT5*-KO cells using an RNA cell miniprep kit (Promega, Fitchburg, WI, USA) according to the manufacturer’s instructions. cDNA synthesis was performed using a GoScript reverse transcription kit (Promega, China) and then used as a qPCR template with FASTStart essential DNA green master mix (Roche, Basel, Switzerland). The primer sequences were as follows: SIRT5 forward 5′-CGGCCAAGTTCAAGTATGGCA-3′ and reverse 5′-TTTCTGCACTAACACCAGCTC-3′.

**Stable isotope labeling by amino acids in cell culture**

For stable isotope labeling by amino acids in cell culture (SILAC) experiments, PC-3 and PC-3M cells were labeled with light or heavy amino acids in SILAC RPMI 1640 medium over seven growth passages. SILAC RPMI 1640 medium was supplemented with 48 mg/l lysine and 200 mg/l arginine (SILAC “light”) or ^13^C_6_^15^N_2_-lysine and ^13^C_6_^15^N_4_-arginine (SILAC “heavy”). Biological replicates were harvested 2 weeks apart from the identical cell culture. After confirming that the SILAC labeling efficiency was > 98%, the cells were lysed with RIPA buffer, and lysates were quantified by BCA assay. We mixed the two cell lines equally (300 μg each) and performed reduction and alkylation using 15 mM dithiothreitol (DTT) and 60 mM iodoacetamide (IAA). Overnight trypsin digestion at 37 °C was used to digest proteins into peptides. Then, high-pH reverse-phase fractionation and off-gel fractionation were performed according to the manufacturer’s protocol.

**Sample preparation for proteomic analysis**

**For sample preparation and in-solution tryptic digestion, dishes of SILAC-labeled cancer cells were washed twice with PBS on ice and then scraped into 4% SDS lysis buffer with halt protease inhibitor cocktail, 4 M sodium butyrate, and 2 M nicotinamide as Histone deacetylase (HDAC) inhibitors. To ensure complete protein extraction, the cell lysates were disrupted by sonication at 4 °C for 1 min and heated for 5 min at 98 °C. Any unbroken cells were removed by centrifugation at 16,000 *g* f at 4 °C or 10 min, and the supernatant was transferred to a low-protein binding E-tube. Proteins from PC-3 and PC-3M cells were measured using a BCA protein assay kit (**Thermo Fisher Scientific)**. The same amount of proteins from PC-3 and PC-3M cells were carefully mixed for SILAC-based quantitative proteomics. The combined proteins were sequentially reduced and alkylated with 15 mM DTT at 56 °C for 30 min and 60 mM IAA for 30 min at RT in the dark. The detergent and chemical reagents present in the protein mixture were removed after protein precipitation with 10% trichloroacetic acid at 4 °C for 4 h. After centrifugation at 12,000 *g* at 4 °C for 10 min, the protein pellets were washed twice with 20 °C acetone and then resuspended in 50 mM ABC buffer using sonication for 5 min on ice. To generate the peptides, the SILAC proteins were digested with trypsin at 37 °C overnight on a rotator. A 10% solution of TFA was added to the peptides to a final concentration of 1% to stop the trypsin reaction. The reaction mixture was centrifuged at 16,000 *g* for 5 min to remove the enzyme and any undigested protein. Before immunoprecipitation to assess lysine succinylation, the peptides were cleaned using a C18 Sep-Pak according to the manufacturer’s instructions. The C18 3cc Sep-Pak was wetted with 3 ml of 100% Acetonitrile (ACN) and 2 ml of 50% ACN. For equilibration, the Sep-Pak was washed three times by adding 2 ml water containing 0.1% trifluoroacetic acid (TFA). The peptides were loaded slowly into the cartridge and then desalted three times using 2 ml water containing 0.1% TFA. The samples were eluted twice with 1 ml of 75% ACN containing 0.1% TFA each time and for a third time to maximize peptide recovery. The purified samples were dried using a speed-vacuum system. A quantitative colorimetric peptide assay was used to measure the amount of peptide in the samples.**

**LC-MS/MS analysis**

**For peptide fractionation for in-depth quantitative proteomics, to improve protein identification, the SILAC peptides were separated by two different processes, high-pH reverse-phase fractionation, and 3100 OFFGEL fractionation. The fractionation of peptides was performed according to the manufacturer’s procedures. Briefly, peptides (100 μg) were made of 0.1% TEA in ACN and separated using high-pH reverse-phase fractionation kit using eight different elution buffers (5%, 7.5%, 10%, 12.5%, 15%, 17.5%, 20%, and 50% ACN in 0.1% TFA). The eight samples were combined into four samples (*e.g.*, fractions 1 and 5, 2 and 6, *etc.*). Peptides (200 μg) were separated by OFFGEL fractionation using high-resolution 24-well frame IPG strips (pH 3–10). These were also combined into 12 samples derived from 24 fractions. All fractionated samples were desalted using a C18 zip tip and completely dried using a speed-vacuum system before further analysis.**

**All samples were dissolved in solvent A [98% water in 0.1% formic acid (FA)]. In particular,** immunoprecipitation (IP) **samples were centrifuged at 16,000 *g* for 5 min to remove any remaining beads. Samples were analyzed using a high-resolution, accurate mass spectrometry (MS) connected to the Eksigent nanoLC system at mass spectrometry convergence research center. Peptide separation was conducted using a home-made C12 reverse-phase analytical column with a linear gradient of 0%–23% solvent B (100% ACN in 0.1% FA) for 95 min, 23%–90% solvent B for 9 min, and 90% solvent B for 6 min at a sustained flow rate of 300 nl/min. The LTQ-velos Orbitrap was operated in the top 20 data-dependent acquisition (DDA) mode. MS data were collected using the following settings: electrospray source voltage 1.8 kV and a 300°C capillary temperature; FTMS 300–1800 m/z range with 60,000 resolution (*m/z* 400); collision-induced dissociation mode at 28% normalized collision energy (NCE) and an isolation width of 1.7 *m/z*; and 500 minimum signals were required for DDA. Lock mass ion from ambient air (*m/z* 445.120024) was enabled to improve mass accuracy.**

**Bioinformatics**

**Data analysis and bioinformatics for the succinylome were performed as follows. To identify proteins and Ksucc proteins, MS/MS spectra were processed using MaxQuant 1.5.1.0 to query the UniProtKB human database (including 71,772 protein sequences; 19029910). The search parameters used a full mass error of 20 ppm and an MS/MS error of 0.5 Da. Trypsin/p was used as the digestion enzyme, allowing for two missing cleavages. The fixed modification was carbamidomethylation of cysteine. For the proteomic search, the oxidation of methionine and acetylation of the N-terminus were set as the variable modifications. For succinylation analysis, Ksu was added as the variable modification. For quantification by SILAC, Lys8 (^13^C_6_^15^N_2_) and Arg10 (^13^C_6_^15^N_4_) were specified as the heavy labels (H), and Lys0 (^12^C_6_^14^N_2_) and Arg0 (^12^C_6_^14^N_4_) were set as the light labels (L). The SILAC pairs (H/L) were detected and quantified from full MS using MaxQuant software. The ratio determined the H/L and 2 of the minimum ratio count. The other parameters for MaxQuant were set to default values.**

**The search results were filtered with a false discovery rate below 0.01, a MaxQuant score more than 40, discarding potential contaminants, and those only identified by a site modification. Also, Ksu sites were specified using a site localization probability > 0.75. It should be noted that the Ksucc ratio was normalized to protein levels. All ratios of proteins and lysine succinylated peptides were converted to a log_2_ scale.**

**Protein co-immunoprecipitation**

LDHA (Cell Signaling Technology, Catalog No. 2012S), SIRT5 (Abcam, Catalog No. 154082) or mouse IgG (Santa Cruz Biochemicals, Dallas, TX, Catalog No. SC-2025) antibody, and protein G magnetic nanobeads (Bioneer, Daejeon, Korea, Catalog No. TA-1021-1) were mixed and incubated in a rotator for 30 min at RT and then washed with nanobeads twice. At least 500 ng of protein were mixed with nanobeads and then incubated in a rotator for 1 h at RT and then washed with nanobeads twice. The elution buffer was added and eluted in a vortex for 5–10 min and then incubated for 10 min in 4% SDS at 95 °C.

**Indirect ELISA for determination of LDHA-K118su in PCa tissues**

Prostate tissue was lysed with RIPA buffer (Thermo), and 1 μg tissue sample was mixed with 50 μl coating buffer (0.2 M sodium bicarbonate, pH 9.4) and incubated for 7 h at RT in a 96-well plate (Corning). Subsequently, these samples were washed three times with wash buffer (TBS-T, pH 7.2) and incubated overnight at 4 °C in blocking buffer (3% BSA in TBS-T). Primary antibodies against LDHA-K118su (CTM-212; PTM Biolabs) were added to each well with a dilution of 1:1000. After 7 h of incubation, these samples were washed three times with wash buffer. Next, HRP-conjugated rabbit antibody was added to each well (1:2000) and incubated for 3 h and then washed five times. TMB substrate solution (Thermo Fisher Scientific) was added to each well. After 10 –15 min, when the color was changed properly, a stop solution (Thermo Fisher Scientific) was added, and OD values at 450 nm were recorded.
