# Supplementary material for: LDHA Desuccinylase Sirtuin 5 as A Novel Cancer Metastatic Stimulator in Aggressive Prostate Cancer
Source: Genomics Proteomics Bioinformatics. 2022 Mar 9;21(1):177–89. doi: 10.1016/j.gpb.2022.02.004 (PMC10372916; doi:10.1016/j.gpb.2022.02.004)
Supplement: Supplementary Table S1 [file mmc2.docx]

**Table S1 Relationship between clinical characteristics and SIRT5 expression in PCa patients**

| **Variables** | **Category** | **No. of patients** | **SIRT5 level** | | ***P* value** |
| --- | --- | --- | --- | --- | --- |
|  |  |  | **High** | **Low** |  |
| Age (yr) | < 65 | 13 | 4 | 9 | 0.209 |
|  | > 66 | 12 | 7 | 5 |  |
| Weight (kg) | < 68 | 11 | 4 | 7 | 0.674 |
|  | > 69 | 14 | 7 | 7 |  |
| BMI | < 25 | 9 | 5 | 4 | 0.903 |
|  | > 26 | 15 | 8 | 7 |  |
| ECOG performance | Yes | 13 | 7 | 5 | 0.014 |
|  | No | 12 | 4 | 8 |  |
| Drinking | Yes | 9 | 7 | 2 | 0.151 |
|  | No | 15 | 4 | 11 |  |
| Smoking | Yes | 11 | 3 | 8 | 0.01 |
|  | No | 14 | 8 | 6 |  |
| Voiding difficulty | Yes | 13 | 10 | 3 | 0.318 |
|  | No | 11 | 3 | 8 |  |
| **pT** | **T2** | **11** | **7** | **4** | **0.003** |
|  | **T3** | **12** | **4** | **8** |  |
| PSA | < 9 | 14 | 9 | 5 | 0.013 |
|  | > 9 | 11 | 2 | 9 |  |
| Gleason score | 6–7 | 13 | 6 | 7 | 0.596 |
|  | > 8 | 9 | 4 | 5 |  |

*Note*: Letter in bold indicate the most significant correlation between SIRT5 expression level in clinical cancer tissues and clinical indicator tumor stages. PCa, prostate cancer; ECOG, European Cooperative Oncology Group; BMI, body mass index; PSA, prostate-specific antigen.
