# Supplementary material for: LDHA Desuccinylase Sirtuin 5 as A Novel Cancer Metastatic Stimulator in Aggressive Prostate Cancer
Source: Genomics Proteomics Bioinformatics. 2022 Mar 9;21(1):177–89. doi: 10.1016/j.gpb.2022.02.004 (PMC10372916; doi:10.1016/j.gpb.2022.02.004)
Supplement: Supplementary Table S3 [file mmc4.docx]

**Table S3. Number of lysine succinylation sites and proteins in PCa cells.**

| Succinylome | Identified | Quantified | Normalized quantification | **PC-3M vs PC-3 cells** | |
| --- | --- | --- | --- | --- | --- |
|  |  |  |  | Up-regulated | Down-regulated |
| Ksu proteins | 169 | 156 | 136 (80.5%) | 64 (41.0%) | 1 (0.6%) |
| Ksu peptides | 442 | 427 | 403 (91.2%) | 144 (33.7%) | 1 (0.2%) |
| Ksu sites | 448 | 430 | 406 (90.6%) | 144 (33.4%) | 1 (0.2%) |
| Proteomics | 3396 | 2958 (87.1%) | — | 36 (1.2%) | 158 (5.3%) |

***Note*: Ksu, lysine succinylation. %, a percentage of the quantified number**
