# Supplementary material for: LDHA Desuccinylase Sirtuin 5 as A Novel Cancer Metastatic Stimulator in Aggressive Prostate Cancer
Source: Genomics Proteomics Bioinformatics. 2022 Mar 9;21(1):177–89. doi: 10.1016/j.gpb.2022.02.004 (PMC10372916; doi:10.1016/j.gpb.2022.02.004)
Supplement: Supplementary Table S4 [file mmc5.docx]

**Table S4 List of up-regulated Ksu proteins, associated with the extracellular and cytoplasm terms, in PC-3M cells**

| **Protein ID** | **Gene name** | **Protein**  **Score** | **Modified sequence** | **Position in protein** | **Ksu ratio**  **(Log2)** | **Protein ratio**  **(Log2)** | **Normalized Ksu ratio**  **(Log2)** | **Ratio H/L count** |
| --- | --- | --- | --- | --- | --- | --- | --- | --- |
| P00338 | *LDHA* | 217.9 | _(ac)ATLK(su)DQLIYNLLK_ | 5 | 1.296 | −0.050 | 1.345 | 1 |
| P00338 | *LDHA* | 72.2 | _TPK(su)IVSGK_ | 76 | 1.334 | −0.050 | 1.383 | 2 |
| P00338 | *LDHA* | 152.4 | _NVNIFK(su)FIIPNVVK_ | 118 | 1.003 | −0.050 | 1.053 | 3 |
| P00338 | *LDHA* | 63.3 | _EVHK(su)QVVESAYEVIK_ | 232 | 1.172 | −0.050 | 1.222 | 1 |
| P00558 | *PGK1* | 125.7 | _LTLDK(su)LDVK_ | 11 | 1.177 | 0.163 | 1.014 | 3 |
| P00558 | *PGK1* | 79.5 | _IVK(su)DLMSK_ | 267 | 1.360 | 0.163 | 1.197 | 3 |
| P00558 | *PGK1* | 105.6 | _GTK(su)ALMDEVVK_ | 353 | 1.245 | 0.163 | 1.082 | 1 |
| P00558 | *PGK1* | 98.0 | _ALMDEVVK(su)ATSR_ | 361 | 1.334 | 0.163 | 1.172 | 3 |
| P04075 | *ALDOA* | 89.1 | _K(su)ELSDIAHR_ | 14 | 1.879 | 0.521 | 1.358 | 2 |
| P04075 | *ALDOA* | 120.9 | _VDK(su)GVVPLAGTNGETTTQGLDGLSER_ | 111 | 1.551 | 0.521 | 1.029 | 2 |
| P07355 | *ANXA2* | 165.2 | _TPAQYDASELK(su)ASMK_ | 115 | 1.526 | 0.473 | 1.053 | 1 |
| P07355 | *ANXA2* | 106.4 | _TDLEK(su)DIISDTSGDFRK_ | 157 | 1.581 | 0.473 | 1.108 | 3 |
| P07355 | *ANXA2* | 67.5 | _K(su)LMVALAK_ | 169 | 1.671 | 0.473 | 1.198 | 1 |
| P07737 | *PFN1* | 94.3 | _TK(su)STGGAPTFNVTVTK_ | 91 | 0.787 | −0.398 | 1.184 | 1 |
| P07737 | *PFN1* | 148.5 | _STGGAPTFNVTVTK(su)TDK_ | 105 | 0.659 | −0.398 | 1.057 | 2 |
| P07737 | *PFN1* | 42.8 | _K(su)CYEMASHLR_ | 127 | 0.697 | −0.398 | 1.095 | 2 |
| P08670 | *VIM* | 126.4 | _FANYIDK(su)VR_ | 120 | 0.832 | −0.383 | 1.215 | 3 |
| P08670 | *VIM* | 127.8 | _K(su)VESLQEEIAFLK_ | 223 | 0.731 | −0.383 | 1.114 | 1 |
| P08670 | *VIM* | 124.4 | _TLLIK(su)TVETR_ | 445 | 1.038 | −0.383 | 1.420 | 3 |
| P27797 | *CALR* | 60.2 | _NVLINK(su)DIR_ | 159 | 0.716 | −0.927 | 1.643 | 1 |
| P30041 | *PRDX6* | 63.6 | _VVFVFGPDK(su)K_ | 141 | 1.391 | 0.338 | 1.053 | 1 |
| P62937 | *PPIA* | 86.4 | _VSFELFADKVPK(su)TAENFR_ | 31 | 0.670 | −0.392 | 1.061 | 3 |
| P62937 | *PPIA* | 105.7 | _ALSTGEK(su)GFGYK_ | 44 | 1.064 | −0.392 | 1.456 | 3 |
| P62937 | *PPIA* | 48.6 | _TEWLDGK(su)HVVFGK_ | 125 | 1.041 | −0.392 | 1.433 | 1 |
| P62937 | *PPIA* | 110.2 | _VK(su)EGMNIVEAMER_ | 133 | 0.929 | −0.392 | 1.321 | 2 |
| P68032 | *ACTC1* | 79.5 | _GILTLK(su)YPIEHGIITNWDDMEK_ | 70 | 0.992 | −0.211 | 1.203 | 1 |
| P68032 | *ACTC1* | 75.8 | _YPIEHGIITNWDDMEK(su)IWHHTFYNELR_ | 86 | 1.202 | −0.211 | 1.413 | 4 |
| P68363 | *TUBA1B* | 77.4 | _TIGGGDDSFNTFFSETGAGK(su)HVPR_ | 60 | 1.169 | −0.163 | 1.333 | 3 |
| P68363 | *TUBA1B* | 89.5 | _GDVVPK(su)DVNAAIATIK_ | 326 | 0.941 | −0.163 | 1.104 | 3 |
| P68363 | *TUBA1B* | 134.4 | _DVNAAIATIK(su)TK_ | 336 | 0.885 | −0.163 | 1.048 | 3 |
| P68363 | *TUBA1B* | 121.3 | _LDHK(su)FDLMYAK_ | 394 | 1.128 | −0.163 | 1.292 | 2 |
| P68363 | *TUBA1B* | 112.4 | _FDLMYAK(su)R_ | 401 | 1.143 | −0.163 | 1.306 | 2 |
| Q562R1 | *ACTBL2* | 75.6 | _IK(su)IIAPPER_ | 329 | 0.732 | −0.462 | 1.194 | 4 |

**Ksu and protein ratios were expressed as log2-transformed changes in PC-3 versus PC-3M cells.**
