# Supplementary material for: LDHA Desuccinylase Sirtuin 5 as A Novel Cancer Metastatic Stimulator in Aggressive Prostate Cancer
Source: Genomics Proteomics Bioinformatics. 2022 Mar 9;21(1):177–89. doi: 10.1016/j.gpb.2022.02.004 (PMC10372916; doi:10.1016/j.gpb.2022.02.004)
Supplement: Supplementary Figure S5 [file mmc6.pptx]

## Slide 1
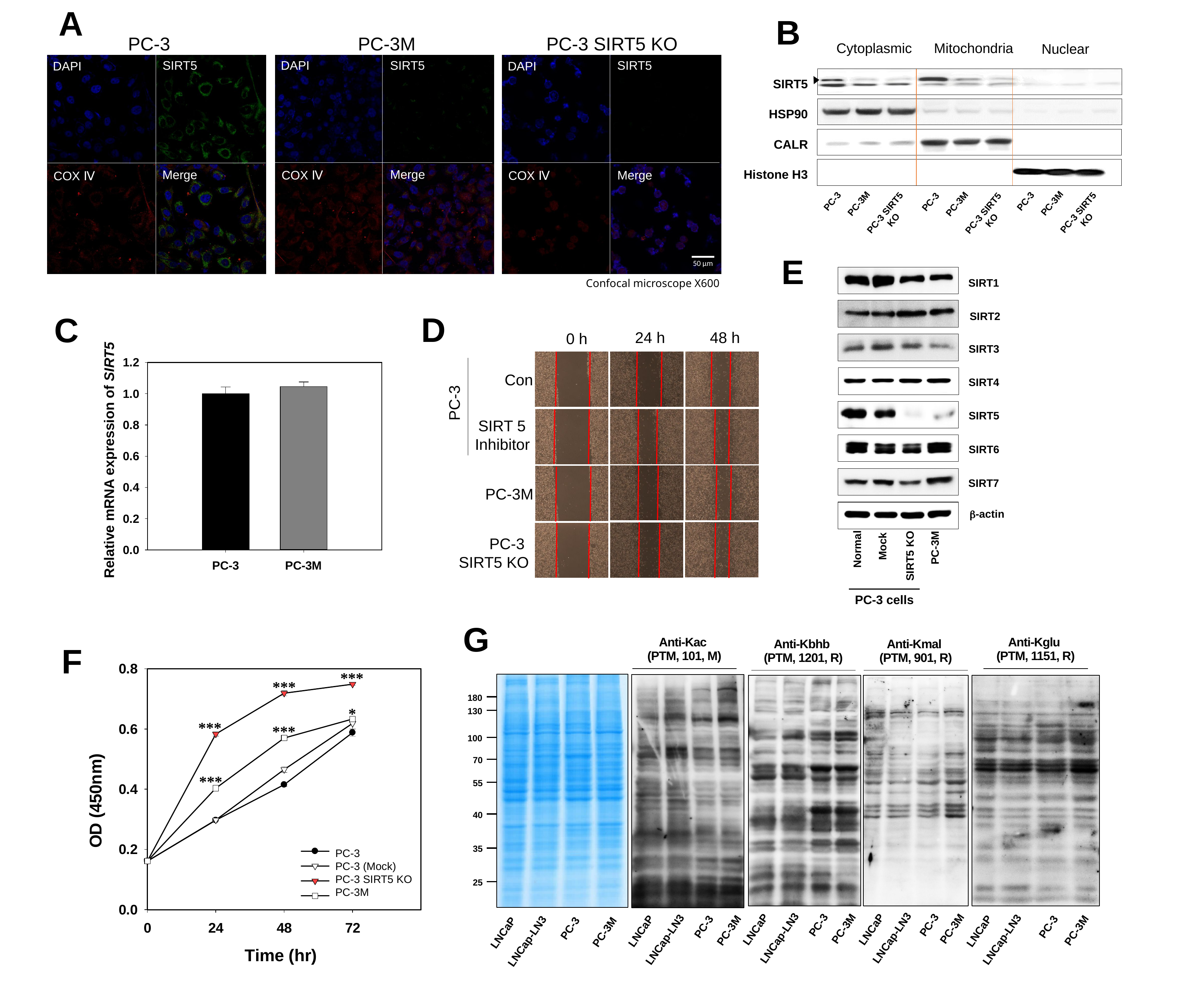

A
B
PC-3
PC-3M
PC-3 SIRT5 KO
Cytoplasmic
Mitochondria
Nuclear
SIRT5
HSP90
CALR
Histone H3
PC-3M
PC-3M
PC-3M
PC-3
PC-3
PC-3
PC-3 SIRT5 KO
PC-3 SIRT5 KO
PC-3 SIRT5 KO
SIRT5
SIRT5
DAPI
SIRT5
DAPI
DAPI
Merge
Merge
Merge
COX Ⅳ
COX Ⅳ
COX Ⅳ
E
50 μm
SIRT1
SIRT2
SIRT3
SIRT4
SIRT5
SIRT6
SIRT7
b-actin
Normal
SIRT5 KO
PC-3M
Mock
PC-3 cells
Confocal microscope X600
D
C
Relative mRNA expression of SIRT5
24 h
48 h
0 h
Con
PC-3
SIRT 5
Inhibitor
PC-3M
PC-3
SIRT5 KO
G
| Anti-Kac (PTM, 101, M) |
| --- |
| Anti-Kglu (PTM, 1151, R) |
| --- |
| Anti-Kbhb (PTM, 1201, R) |
| --- |
| Anti-Kmal (PTM, 901, R) |
| --- |
F
***
***
*
***
***
***
180
130
100
70
55
40
35
25
PC-3
PC-3 (Mock)
PC-3 SIRT5 KO
PC-3M
PC-3
LNCaP
PC-3M
LNCap-LN3
PC-3
LNCaP
PC-3M
LNCap-LN3
PC-3
LNCaP
PC-3M
LNCap-LN3
PC-3
LNCaP
PC-3M
LNCap-LN3
PC-3
LNCaP
PC-3M
LNCap-LN3
