# Supplementary material for: LDHA Desuccinylase Sirtuin 5 as A Novel Cancer Metastatic Stimulator in Aggressive Prostate Cancer
Source: Genomics Proteomics Bioinformatics. 2022 Mar 9;21(1):177–89. doi: 10.1016/j.gpb.2022.02.004 (PMC10372916; doi:10.1016/j.gpb.2022.02.004)
Supplement: Supplementary Figure S6 [file mmc7.pptx]

## Slide 1
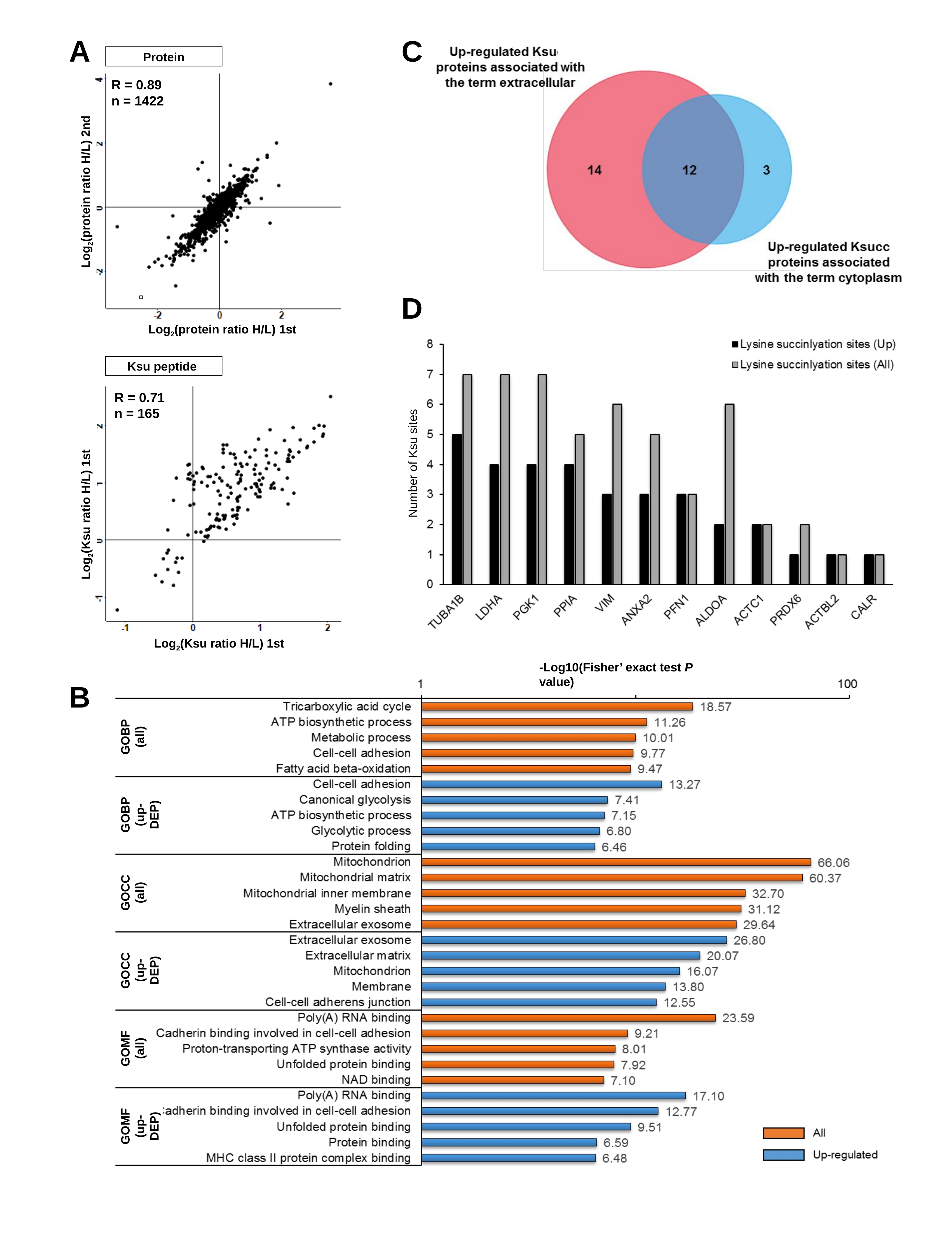

A
C
Protein
R = 0.89
n = 1422
Log2(protein ratio H/L) 2nd
D
Log2(protein ratio H/L) 1st
Ksu peptide
R = 0.71
n = 165
Number of Ksu sites
Log2(Ksu ratio H/L) 1st
Log2(Ksu ratio H/L) 1st
-Log10(Fisher’ exact test P value)
B
GOBP
(all)
GOBP
(up-DEP)
GOCC
(all)
GOCC
(up-DEP)
GOMF
(all)
GOMF
(up-DEP)
