# Supplementary figures and images for: LDHA Desuccinylase Sirtuin 5 as A Novel Cancer Metastatic Stimulator in Aggressive Prostate Cancer
Source: Genomics Proteomics Bioinformatics. 2022 Mar 9;21(1):177–89. doi: 10.1016/j.gpb.2022.02.004 (PMC10372916; doi:10.1016/j.gpb.2022.02.004)

## Slide 1
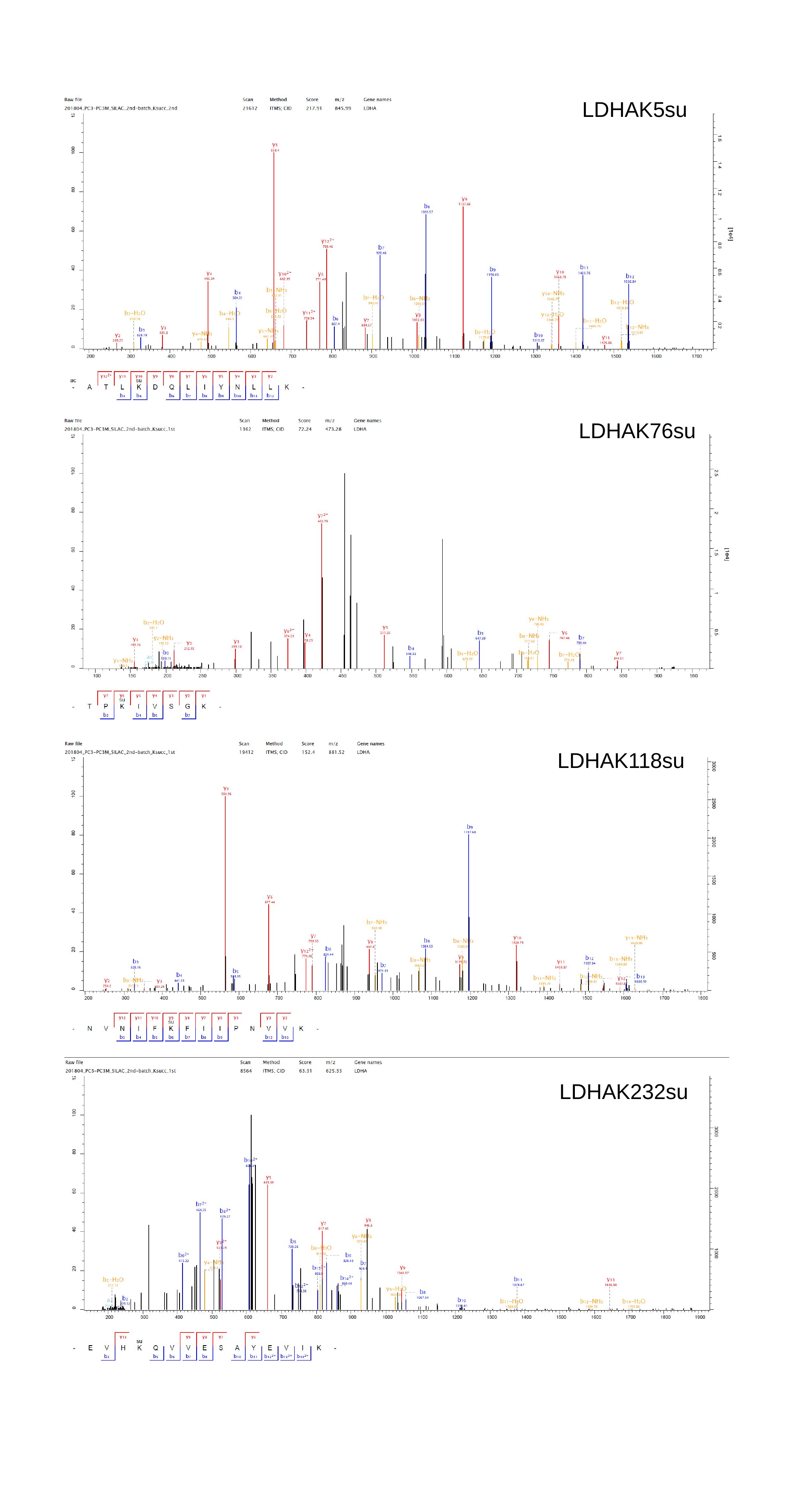

LDHAK5su
LDHAK76su
LDHAK118su
LDHAK232su

Supplement: Supplementary Figure S7 [file mmc8.pptx]
